# Therapeutic interventions for alcohol dependence in non-inpatient settings: a systematic review and network meta-analysis (protocol)

**DOI:** 10.1186/s13643-017-0462-2

**Published:** 2017-04-11

**Authors:** Hung-Yuan Cheng, Roy G. Elbers, Julian P. T. Higgins, Abigail Taylor, Georgina J. MacArthur, Luke McGuinness, Sarah Dawson, José A. López-López, Sean Cowlishaw, Matthew Hickman, David Kessler

**Affiliations:** 1 School of Social and Community Medicine, University of Bristol, Canynge Hall, 39 Whatley Road, Bristol, BS8 2PS UK; 2 National Institute for Health Research (NIHR) Health Protection Research Unit (HPRU) on Evaluation of Interventions, University of Bristol, Bristol, UK; 3 NIHR School of Primary Care Research, Bristol, UK

**Keywords:** Alcohol dependence, Detoxification, Network meta-analysis, Systematic review

## Abstract

**Background:**

Alcohol dependence is common and serious cause of social and physical harm. However, the optimal management of those with moderate and severe alcohol dependence in primary and community care after detoxification remains unclear. The aim of this review is to evaluate the effectiveness of interventions for maintaining abstinence in people with alcohol dependence following detoxification.

**Methods:**

We will systematically search electronic databases and clinical trial registries for randomized controlled trials (RCTs) examining the effectiveness of pharmacological and/or psychosocial interventions for maintaining abstinence in recently detoxified, alcohol-dependent adults. The searches will be complemented by checking references and citations from included studies and other relevant systematic reviews. No limitation on language, year, or publication status will be applied. RCTs will be selected using prespecified criteria. Descriptive information, study characteristics, and results of eligible RCTs will be extracted. A revised version of the Cochrane Risk of Bias tool (RoB 2.0) will be used to assess the risk of bias in eligible RCTs. Results will be synthesized and analyzed using network meta-analysis (NMA). Overall strength of the evidence and publication bias will be evaluated. Subgroup and sensitivity analysis will also be performed.

**Discussion:**

This network meta-analysis aims to appraise and summarize the total evidence of therapeutic interventions for alcohol-dependent patients that require support for detoxification and can be treated in the community. The evidence will determine which combination of interventions are most promising for current practice and further investigation.

**Systematic review registration:**

PROSPERO CRD42016049779

**Electronic supplementary material:**

The online version of this article (doi:10.1186/s13643-017-0462-2) contains supplementary material, which is available to authorized users.

## Background

Alcohol dependence is a long-term problematic pattern of alcohol use leading to serious negative health outcomes. Chronic and excessive alcohol use has been associated with increased risk of cancer, diabetes, infectious disease, cardiovascular disease, and neuropsychiatric disease [1]. The negative effects of alcohol on cognitive processing and behavior also contribute to psychosocial problems [2–4]. The consequence of these is a large cost to social and healthcare systems [5]. Within the UK alone, it is estimated that alcohol-related harm and prescription items for treating alcohol dependence cost around £21 and £4 billion per year, respectively [6, 7].

Management of alcohol dependence is challenging. In the UK, only about 60% of alcohol-dependent patients are completely free of dependency from alcohol after treatment [8]. This indicates scope for improvement in clinical outcomes. Many patients drop out of treatment, particularly those exhibiting severe alcohol dependence and comorbidities. This is not only wasteful of resources, but clinically risky as repeated withdrawals from alcohol are associated with an increase in the severity of withdrawal symptoms and can precipitate poor outcomes [9].

There is a growing trend away from inpatient to outpatient treatment for alcohol-dependent patients [8]. For patients with moderate and severe dependence, alcohol detoxification is a crucial part of management. While the evidence supporting community detoxification is robust [10–14], there is an evidence gap relating to continuing care for detoxified patients in order to maintain abstinence. Pharmacological interventions including acamprosate, naltrexone, and disulfiram, in conjunction with psychotherapy, have been recommended [15]. However, it is not clear what works best in non-inpatient settings, such as primary and community care.

Several reviews have been undertaken of the effectiveness of therapeutic interventions on maintaining abstinence in detoxified alcohol-dependent patients [16–23]. Their conclusions were based on subgroup analyses of pairwise meta-analyses of specific interventions, such as acamprosate [16–22], naltrexone [16, 20], and sodium oxybate [20, 21, 23]. However, the comparative efficacy of these and other interventions has not been established. Moreover, the effects of conjunct psychotherapy on the efficacy of interventions are underexplored. To date, it is unclear how effective are pharmacotherapy and psychotherapy in maintaining abstinence for alcohol-dependent adults in non-inpatient settings following detoxification. The aim of this systematic review is thus to appraise and summarize the total evidence of therapeutic interventions for detoxified alcohol-dependent patients in primary and community care settings.

## Methods

Methods are derived from the Cochrane Handbook for Systematic Reviews of Interventions [24]. The reported items are in compliance with the Preferred Reporting Items for Systematic Reviews and Meta-Analysis Protocol (PRISMA-P) [25]. The PRISMA-P checklist can be found in Additional file 1. This review protocol has been registered with the International Prospective Register of Systematic Reviews (PROSPERO) on 25 October 2016 (registration number CRD42016049779). Any amendments to this protocol will be described in the final review.

### Eligibility criteria

#### Study design

We will include both individualized and cluster randomized controlled trials (RCTs).

#### Population

Eligible populations are adults aged over 18 years with alcohol dependence, diagnosed using standardized diagnostic criteria (e.g., Diagnostic and Statistical Manual of Mental Disorders (DSM), International Classification of Diseases (ICD)), or the Alcohol Use Disorders Identification Test (AUDIT; score ≥20), who have undergone detoxification in less than 4 weeks. Studies including pregnant women will be excluded.

#### Interventions/comparators

We will include any therapeutic intervention for maintaining abstinence in alcohol-dependent adults. Interventions should be applicable to non-inpatient settings (such as primary care and community settings). The following interventions will be considered but the list is not exhaustive and interventions will be added when identified through searches:

1. Opioid antagonists: naltrexone, nalmefene
2. Anticonvulsants: valproates, pregabalin, topiramate, levetiracetam, oxcarbazepine, gabapentin
3. Antidepressants: sertraline, fluoxetine, escitalopram, paroxetine, citalopram, desipramine
4. Antipsychotics: olanzapine, tiapride, aripiprazole
5. Alcohol deterrents: disulfiram, acamprosate, calcium carbimide
6. Miscellaneous agents: baclofen, sodium oxybate, ondansetron, varenicline, nefazodone
7. Psychotherapies: cognitive behavioral therapy (CBT), motivational enhancement therapy (MET), 12-step facilitation therapy, coping skills, motivational interviewing
8. Concomitant or adjunctive therapies: naltrexone and CBT, acamprosate and CBT, naltrexone and sodium oxybate

We aim to compare interventions with placebo or other interventions using network meta-analysis (NMA) within the frequentist framework.

#### Outcomes

The primary outcome is abstinence at least 12 weeks after randomization. There are two main reasons for this. First, abstinence is the most appropriate and commonly reported outcome for severely dependent drinkers. Second, completely abstaining from alcohol has been shown to improve cognitive function [26, 27] and quality of life [28–30].

Secondary outcomes that will be considered are (1) amount of alcohol consumption, (2) drinking frequency, (3) intervention compliance, (4) adverse events, and (5) withdrawal from study.

Depending on the length of follow-up reported, the end-points for each outcome will be categorized into short-term (12 to 20 weeks), intermediate-term (20 to 36 weeks), and long-term (more than 36 weeks). Longer follow-up periods will also be considered.

### Search strategy

Electronic searches will be conducted using the following bibliographic databases: Cochrane Central Register of Controlled Trials (CENTRAL), Ovid Embase, MEDLINE, and PsycINFO. The search will start from inception of each database and be updated toward the end of the review.

Search strategies will be developed and applied by an information specialist. These strategies will be based on a combination of controlled vocabulary and keywords according to the Cochrane Handbook [31]. We will not restrict search strategies by language, date, or publication status. The strategy developed for Ovid MEDLINE will be adapted for other databases. The Ovid MEDLINE search strategy can be found in Additional file 2. Reference lists and citations from all included studies will be hand-searched to identify further studies.

Reference lists from other systematic reviews and meta-analyses will be examined to identify further eligible studies. The reviews will be identified from the primary searches used to identify RCTs, the Cochrane Database of Systematic Reviews (CDSR) and Epistemonikos. We will also conduct a separate search on Ovid MEDLNE using a systematic review filter.

Trial registries (i.e., ClinicalTrials.gov, EU Clinical Trials Register (EudraCT), and the World Health Organization International Clinical Trials Registry Platform (ICTRP)) will be searched to identify relevant registered trials and reports.

### Study selection

Search results will initially be managed using Endnote. A bespoke Excel spreadsheet will be used for study selection against a predefined set of inclusion and exclusion criteria. The selection of references will go through two screening processes: an initial screening and a full-text examination. During the initial screening, a team of six reviewers will independently screen titles and abstracts using the inclusion and exclusion criteria to remove irrelevant references. At least a third of references will be dual-screened. Any disagreement between reviewers will be resolved by discussion or consultation with a third reviewer. The consistency of agreement amongst reviewers is maintained by pairing the first author with other reviewers in the majority of the initial screening. If there is insufficient information in the title and abstract to make a decision, the full-text will be retrieved. References thought to be potentially relevant in languages not known by reviewers will be translated, and initial screening will take place using the same procedure.

Studies passed through initial screening will then be subject to full-text examination. In this stage, four reviewers will be involved in identification of eligible studies using an independent, dual-screening approach. The first author will pair with other reviewers to dual-screen full-text, to minimize interrater variation. Any disagreement between pairs will be resolved by discussion or, if unresolved, by consulting a senior author. Reasons for studies excluded during this stage will be documented and reported. A PRISMA flow diagram will be used to document the flow of records [32].

### Data extraction

Data from studies that meet the inclusion criteria and with sufficient information of study description, participant characteristics, definitions of outcome measures, and outcome results will be extracted independently by two reviewers using a pilot-tested data extraction form. The first author will extract information from all reports, pairing with one of three other reviewers to dual-extract data. Any disagreement will be discussed and, if necessary, a senior author will be consulted to achieve consensus. When there are multiple publications from the same study, all publications will be examined and only the most comprehensive or up-to-date information will be extracted to ensure that the study is not over-represented in the review.

Data extracted from each study will include the following:

1. General information (e.g., year, lead author, study title, unique identifier from ClinicalTrials.gov or equivalent)
2. Study information (e.g., country, setting, recruitment, screening process, duration, use of diagnostic criteria, inclusion criteria, exclusion criteria)
3. Participant characteristics (e.g., number, age, gender, severity of alcohol dependence, baseline drinking behaviors, social background, primary diagnosis, comorbid diagnoses, concurrent substance abuse)
4. Interventions and comparator (e.g., types, regimes, additional treatment, length)
5. Outcome measures and assessment methods
6. Details for risk of bias assessment (e.g., randomization methods, blinding, intention-to-treat analysis, use of methods for handling missing data and dropout)
7. Source of funding

### Dealing with missing data

We will contact corresponding authors of included studies to obtain any unreported and missing data. Our primary interest is the effect of assignment to intervention, so we will seek results for the intention-to-treat population. If data are missing due to participant drop out, we will use reported results for participants that completed the study. A sensitivity analysis for unreported and missing data will be performed, and any issues will be recorded using the approaches adapted from the Cochrane Handbook [33].

### Assessment of risk of bias

The risk of bias in each study will be assessed using a recently developed revision of the Cochrane risk of bias tool (RoB 2.0: a revised tool to assess risk of bias in randomized trials) [34]. Two pairs of reviewers will independently assess five domains of bias for each outcome. These five domains are bias due to (1) the randomization process, (2) deviations from intended interventions, (3) missing outcome data, (4) measurement of the outcome, and (5) selection of the reported results.

Answers to signaling questions and supporting information will collectively lead to a domain-level judgment in the form of “Low Risk,” “Some Concerns”, or “High Risk” of bias. These domain-level judgements will inform an overall risk of bias judgment for the outcome. Discrepancies between the two reviewers will be resolved by discussion to reach consensus. If necessary, a third reviewer will be consulted to achieve a decision.

For each outcome, we will assess the quality of the evidence of the NMA results using an adapted version of the Grading of Recommendations Assessment, Development and Evaluation (GRADE) methodology [35]. We produce a “summary of findings table” for each clinical recommendation outcome [36].

To assess publication biases, we plan to conduct the assessment through “comparison-adjusted” [37] and “contour-enhanced” [38] funnel plots if we find sufficient studies. If asymmetry is found, we will examine variation of the studies to elucidate whether the cause of asymmetry is likely due to publication bias or a real relationship between trial size and effect.

### Data synthesis

Considering the large body of literature in this topic, we plan to combine and compare all interventions using NMA. We will analyze each identified interventions as a different “node” in the network. Different doses or exposure times of the same pharmacological or psychosocial interventions will be categorized into a small number of nodes according to the length, strength, and clinical consideration. Control groups in studies of pharmacological and psychosocial interventions may be rather different, and we will treat these as separate nodes in the network. For interventions that cannot be included in the NMA model, their results will be summarized and narratively described in the final review if it is possible.

NMA rests on an assumption of transitivity or exchangeability across studies, which implies that any patient in the network could have been assigned to any of the interventions in the network. To maximize the validity of this assumption, we are focusing the review on a specific population (detoxified, alcohol-dependent patients) in a specific setting (community non-inpatient settings). However, we expect that some clinical and methodological variables, including social background, comorbid diagnoses, and co-interventions, may influence the effectiveness or acceptability of the interventions of interest. We plan to investigate the clinical and methodological comparability across interventions to assure transitivity after data extraction [39].

We will generate a table of descriptive characteristics for included studies. Relevant outcome data will be synthesized by using odds ratios (ORs) for dichotomous outcomes and standardized mean differences (SMDs) for continuous measures. The estimated treatment effects from included studies, along with 95% confidence intervals (CIs), will be plotted for each pairwise comparison for inspection of heterogeneity across studies.

We will examine pairwise comparisons for each intervention across studies for each outcome, in order to assess statistical heterogeneity. Heterogeneity variances obtained from these pairwise comparisons will be used to investigate the possibility of statistical heterogeneity. The inconsistency in the NMA, referring to the degree of disagreement between source-specific treatment effects [39], will be evaluated locally by comparing the direct and indirect summary effect estimates across studies using the node-split approach [40] and globally by using the design-by-treatment interaction model [41, 42].

The NMA will be based on random-effects models to account for heterogeneity in treatment effects within each comparison, assuming a common heterogeneity variance (τ^2^) across all comparisons [35]. We intend to perform our NMA model with contrast-level data by using multivariate meta-analysis approaches within the frequentist framework in STATA [43]. We will use the *network* suite of STATA commands [44]. The restricted maximum likelihood method will be used to estimate between-study variance in the NMA. However, this is subject to the data and structure of the network. The final statistical approaches and strategy will be developed by experienced statisticians at a further stage in the review. The summary ORs or SMDs, including the 95% CIs, for all comparisons will be presented in a summary table for each outcome. Furthermore, we will provide ranking probabilities for interventions in rankograms and cumulative ranking probability plots for the primary outcome [45].

### Subgroup and sensitivity analysis

Given the variety of study settings in the literature, we plan to perform subgroup analyses for the primary outcome using random-effects meta-regression approaches [46]. Potential variables used for subgrouping will include length of intervention, optional psychosocial interventions, dosing and schedule of interventions, psychiatric co-morbidity, severity of alcohol dependence, and social background. Sensitivity analyses will be conducted to explore the impact of imputation and risk of bias on outcome data.

## Discussion

This review will assess the comparative effectiveness of therapeutic interventions to maintain abstinence for alcohol-dependent patients following detoxification in community non-inpatient settings. Currently, there is no comprehensive systematic review of interventions across different pharmacological and psychosocial modalities to inform the clinical practice for the treatment of alcohol dependence in detoxified patients. A systematic review specifically bridging the gap between detoxification and alcohol dependence treatment has not been undertaken, to our knowledge. Maisel et al. conducted a meta-analysis to examine effects of different moderators on the efficacy of n altrexone and acamprosate on alcohol use disorders [16]. However, there are limitations to the clinical application of their conclusions as they did not assess risk of bias in the individual studies. Timko et al. identified factors affecting detoxification completion and subsequent treatment in detoxified alcohol-dependent patients [47]. However, their evidence review was based on searches of a single database. Other systematic reviews have applied subgroup analyses to investigate the effect of detoxification on specific interventions [18, 19, 23]. The application of these conclusions is often limited and less likely to be useful for community settings, which present a wide range of patient types and treatment options. The advantage of the network meta-analysis approach is that it offers the opportunity to perform more complex comparisons and compute a ranking effectiveness for interventions. The direct and indirect comparisons evaluating evidences amongst licensed, off-license use medications, and psychosocial interventions enable us to infer appropriate interventions in community settings. Therefore, this evidence will help patients and clinicians to make decisions in such settings. The results will also aid to the development and optimization of new interventions.

## Additional files

Additional file 1: BMC PRISMA-P checklist. (DOCX 30 kb)

Additional file 2: Search Strategy Ovid MEDLINE. (DOCX 16 kb)

## Abbreviations

AUDIT: Alcohol Use Disorders Identification Test
CBT: Cognitive behavioral therapy
CDSR: Cochrane Database of Systematic Reviews
CENTRAL: Cochrane Central Register of Controlled Trials
CI: Confidence interval
DSM: Diagnostic and Statistical Manual of Mental Disorders
EudraCT: EU Clinical Trials Register
GRADE: Grading of Recommendations Assessment, Development and Evaluation
ICD: International Classification of Diseases
ICTRP: World Health Organization International Clinical Trials Registry Platform
MET: Motivational enhancement therapy
NMA: Network meta-analysis
OR: Odds ratio
PRISMA: Preferred Reporting Items for Systematic Reviews and Meta-Analyses
PROSPERO: International Prospective Register of Systematic Reviews
RCT: Randomized controlled trial
RoB 2.0: The revised tool for assessing risk of bias in randomized trials
SMD: Standardized mean difference

## References

[1] Rehm J (2011). The risks associated with alcohol use and alcoholism. Alcohol Res Health.

[2] Uekermann J, Daum I (2008). Social cognition in alcoholism: a link to prefrontal cortex dysfunction?. Addiction.

[3] Gizewski ER, Muller BW, Scherbaum N, Lieb B, Forsting M, Wiltfang J (2013). The impact of alcohol dependence on social brain function. Addict Biol.

[4] Kornreich C, Delle-Vigne D, Knittel J, Nerincx A, Campanella S, Noel X (2011). Impaired conditional reasoning in alcoholics: a negative impact on social interactions and risky behaviors?. Addiction.

[5] Rehm J, Mathers C, Popova S, Thavorncharoensap M, Teerawattananon Y, Patra J (2009). Global burden of disease and injury and economic cost attributable to alcohol use and alcohol-use disorders. Lancet (London, England).

[6] Lifestyles Statistics Team, Health and Social Care Information Centre. In: Statistics on Alcohol: England, 2015. National Statistics. 2015. http://content.digital.nhs.uk/catalogue/pub17712/alc-eng-2015-rep.pdf. Accessed 10 Jan 2017

[7] Lifestyles Statistics Team, Health and Social Care Information Centre. Statistics on Alcohol: England, 2016. National Statistics. 2016. http://content.digital.nhs.uk/catalogue/PUB20999/alc-eng-2016-rep.pdf. Accessed 10 Jan 2017.

[8] Pubilic Health England. Adult substance misuse statistics from the National Drug Treatment Monitoring System (NDTMS). Pubilic Health England. 2016. https://www.ndtms.net/Publications/downloads/Adult%20Substance%20Misuse/adult-statistics-from-the-national-drug-treatment-monitoring-system-2015-2016.pdf. Accessed 10 Jan 2017.

[9] Becker HC (1998). Kindling in alcohol withdrawal. Alcohol Health Res World.

[10] Kattimani S, Bharadwaj B (2013). Clinical management of alcohol withdrawal: a systematic review. Ind Psychiatry J.

[11] Fleeman ND (1997). Alcohol home detoxification: a literature review. Alcohol Alcohol.

[12] Bartu A, Saunders W (1994). Domiciliary detoxification: a cost effective alternative to inpatient treatment. Aust J Adv Nurs.

[13] Feldman DJ, Pattison EM, Sobell LC, Graham T, Sobell MB (1975). Outpatient alcohol detoxification: initial findings on 564 patients. Am J Psychiatry.

[14] Klijnsma MP, Cameron ML, Burns TP, McGuigan SM (1995). Out-patient alcohol detoxification—outcome after 2 months. Alcohol Alcohol.

[15] National Institute for Health and Clinical Excellence. Alcohol-Use Disorders: Diagnosis, Assessment and Management of Harmful Drinking and Alcohol Dependence. NICE Clinical guideline (CG115). Leicester (UK): British Psychological Society; The British Psychological Society & The Royal College of Psychiatrists. 2011. https://www.nice.org.uk/guidance/cg115. Accessed 10 Jan 2017.

[16] Maisel NC, Blodgett JC, Wilbourne PL, Humphreys K, Finney JW. Meta-analysis of naltrexone and acamprosate for treating alcohol use disorders: when are these medications most helpful? Addiction. 2013;108(2):275-93. doi:10.1111/j.1360-0443.2012.04054.x. Epub 2012 Oct 17. 10.1111/j.1360-0443.2012.04054.x PMC3970823 23075288

[17] Mann K, Lehert P, Morgan MY (2004). The efficacy of acamprosate in the maintenance of abstinence in alcohol-dependent individuals: results of a meta-analysis. Alcoholism Clin Exp Res.

[18] Carmen B, Angeles M, Ana M, María AJ (2004). Efficacy and safety of naltrexone and acamprosate in the treatment of alcohol dependence: a systematic review. Addiction.

[19] Rösner S, Hackl-Herrwerth A, Leucht S, Lehert P, Vecchi S, Soyka M. Acamprosate for alcohol dependence. Cochrane Database Syst Rev. 2010;(9):CD004332. doi:10.1002/14651858.CD004332.pub2. 10.1002/14651858.CD004332.pub2 PMC12147086 20824837

[20] Keating GM (2014). Sodium oxybate: a review of its use in alcohol withdrawal syndrome and in the maintenance of abstinence in alcohol dependence. Clin Drug Investig.

[21] Caputo F, Vignoli T, Tarli C, Domenicali M, Zoli G, Bernardi M, et al. A Brief Up-Date of the Use of Sodium Oxybate for the Treatment of Alcohol Use Disorder. Int J Environ Res Public Health. 2016;13(3). doi:10.3390/ijerph13030290. 10.3390/ijerph13030290 PMC4808953 26959045

[22] Boothby LA, Doering PL. Acamprosate for the treatment of alcohol dependence. Clin Ther. 2005;27(6):695–714. 10.1016/j.clinthera.2005.06.015 16117977

[23] Leone MA, Vigna-Taglianti F, Avanzi G, Brambilla R, Faggiano F. Gamma-hydroxybutyrate (GHB) for treatment of alcohol withdrawal and prevention of relapses. Cochrane Database Syst Rev. 2010;17(2):CD006266. doi:10.1002/14651858.CD006266.pub2. 10.1002/14651858.CD006266.pub2 PMC13006865 20166080

[24] Cochrane Handbook for Systematic Reviews of Interventions Version 5.1.0 [updated March 2011] Higgins JPT, Green S, editors. The Cochrane Collaboration; 2011. http://handbook.cochrane.org/. Accessed 10 Jan 2017.

[25] Moher D, Shamseer L, Clarke M, Ghersi D, Liberati A, Petticrew M (2015). Preferred reporting items for systematic review and meta-analysis protocols (PRISMA-P) 2015 statement. Syst Rev.

[26] Kühn S, Charlet K, Schubert F, Kiefer F, Zimmermann P, Heinz A (2014). Plasticity of hippocampal subfield volume cornu ammonis 2+3 over the course of withdrawal in patients with alcohol dependence. JAMA Psychiatry.

[27] Rosenbloom MJ, Rohlfing T, O’Reilly AW, Sassoon SA, Pfefferbaum A, Sullivan EV (2007). Improvement in memory and static balance with abstinence in alcoholic men and women: selective relations with change in brain structure. Psychiatry Res.

[28] Foster JH, Powell JE, Marshall EJ, Peters TJ (1999). Quality of life in alcohol-dependent subjects—a review. Qual Life Res.

[29] Johnson BA, Ait-Daoud N, Akhtar FZ, Ma JZ (2004). Oral topiramate reduces the consequences of drinking and improves the quality of life of alcohol-dependent individuals: a randomized controlled trial. Arch Gen Psychiatry.

[30] Morgan MY, Landron F, Lehert P (2004). Improvement in quality of life after treatment for alcohol dependence with acamprosate and psychosocial support. Alcoholism Clin Exp Res.

[31] Lefebvre C, Manheimer E, Glanville J. Chapter 6: Searching for studies. In: Higgins JPT, Green S, editors. Cochrane Handbook for Systematic Reviews of Interventions Version 5.1.0 [updated March 2011]. The Cochrane Collaboration; 2011 http://handbook.cochrane.org/. Accessed 10 Jan 2017.

[32] Moher D, Liberati A, Tetzlaff J, Altman DG, The PRISMA Group. Preferred reporting items for systematic reviews and meta-analyses: the PRISMA statement. PLoS Med 6(7): e1000097. doi:10.1371/journal.pmed.1000097. 10.1371/journal.pmed.1000097 PMC2707599 19621072

[33] Higgins JPT, Deeks JJ, Altman DG. Chapter 16: Special topics in statistics. In: Higgins JPT, Green S, editors. Cochrane Handbook for Systematic Reviews of Interventions Version 5.1.0 [updated March 2011]. The Cochrane Collaboration; 2011. http://handbook.cochrane.org/. Accessed 10 Jan 2017.

[34] Higgins JPT, Sterne JAC, Savović J, Page MJ, Hróbjartsson A, Boutron I, Reeves B, Eldridge S. A revised tool for assessing risk of bias in randomized trials. In: Chandler J, McKenzie J, Boutron I, Welch V, editors. Cochrane Methods. Cochrane Database of Systematic Reviews. 2016;10(Suppl 1):29-31. http://www.cochranelibrary.com/dotAsset/ecafc5c7-0b9b-4cd1-a4c1-8b0013aea046.pdf.

[35] Salanti G, Del Giovane C, Chaimani A, Caldwell DM, Higgins JPT. Evaluating the Quality of Evidence from a Network Meta-Analysis. PLoS ONE. 2014;9(7):e99682. doi:10.1371/journal.pone.0099682. 10.1371/journal.pone.0099682 PMC4084629 24992266

[36] Puhan MA, Schunemann HJ, Murad MH, Li T, Brignardello-Petersen R, Singh JA (2014). A GRADE Working Group approach for rating the quality of treatment effect estimates from network meta-analysis. BMJ.

[37] Peters JL, Sutton AJ, Jones DR, Abrams KR, Rushton L, Moreno SG (2010). Assessing publication bias in meta-analyses in the presence of between-study heterogeneity. J R Stat Soc Ser A Stat Soc.

[38] Peters JL, Sutton AJ, Jones DR, Abrams KR, Rushton L (2008). Contour-enhanced meta-analysis funnel plots help distinguish publication bias from other causes of asymmetry. J Clin Epidemiol.

[39] Salanti G (2012). Indirect and mixed-treatment comparison, network, or multiple-treatments meta-analysis: many names, many benefits, many concerns for the next generation evidence synthesis tool. Res Synth Methods.

[40] Dias S, Welton NJ, Caldwell DM, Ades AE (2010). Checking consistency in mixed treatment comparison meta-analysis. Stat Med.

[41] White IR, Barrett JK, Jackson D, Higgins JPT (2012). Consistency and inconsistency in network meta-analysis: model estimation using multivariate meta-regression. Res Synth Methods.

[42] Higgins JPT, Jackson D, Barrett JK, Lu G, Ades AE, White IR (2012). Consistency and inconsistency in network meta-analysis: concepts and models for multi-arm studies. Res Synth Methods.

[43] StataCorp (2015). Stata Statistical Software: Release 14.

[44] White IR (2015). Network meta-analysis. Stata J.

[45] Chaimani A, Higgins JPT, Mavridis D, Spyridonos P, Salanti G (2013). Graphical tools for network meta-analysis in STATA. PLoS One.

[46] Deeks JJ, Higgins JPT, Altman DG. Chapter 9: Analysing data and undertaking meta-analyses. In: Higgins JPT, Green S, editors. Cochrane Handbook for Systematic Reviews of Interventions Version 5.1.0 [updated March 2011]. The Cochrane Collaboration; 2011. http://handbook.cochrane.org/. Accessed 10 Jan 2017.

[47] Timko C, Below M, Schultz NR, Brief D, Cucciare MA (2015). Patient and programe factors that bridge the detoxification-treatment gap: a structured evidence review. J Subst Abuse Treat.

