# Supplementary material for: Therapeutic interventions for alcohol dependence in non-inpatient settings: a systematic review and network meta-analysis (protocol)
Source: Syst Rev. 2017 Apr 11;6:77. doi: 10.1186/s13643-017-0462-2 (PMC5387199; doi:10.1186/s13643-017-0462-2)
Supplement: Supplementary file 2 — Search Strategy Ovid MEDLINE. (DOCX 16 kb) [file 13643_2017_462_MOESM2_ESM.docx]

**Ovid MEDLINE Databases**

[Epub Ahead of Print, In-Process & Other Non-Indexed Citations, Ovid MEDLINE(R) Daily and Ovid MEDLINE(R) 1946 to Present]

1. alcohol related disorders/

2. alcoholism/

3. alcohol abstinence/

4. alcoholic intoxication/

5. (alcohol* adj3 (abuse* or addict* or dependen* or disorder* or abstinen*)).ti,ab,kf.

6. alcoholism.ti,kf.

7. (problem* adj2 (drink* or alcohol* use*)).ti,ab,kf.

8. or/1-7

9. exp Narcotic Antagonists/

10. ((Opiate or opioid) and (antagonist* or inhibitor*)).ti,ab,kf,rn.

11. Naltrexone/ or Naloxone/

12. (nalmefene or Revia or Vivitrol or naltrexon or naloxone).ti,ab,kf,rn.

13. exp Dopamine antagonists/

14. exp Antipsychotic Agents/

15. (Dopamine antagonists or (antidopaminergic and (agent* or drug* or intervention* or treatment* or pharmacotherap*))).ti,ab,kf.

16. exp Phenothiazines/

17. (Olanzapine or Zyprexa or asenapine or quetiapine or Seroquel or risperidone or Risperidal or Risperdal or ziprazidone or ziprasidone or aripiprazole or Abilify or Thorazine or Aminazine or haldol or Largactil or Chlordelazine or Chlorpromazine or Contomin or Fenactil or Propaphenin or Chlorazine or Thioridazine* or Thiozine or Tiapride or Rideril or Sonapax or Meleril or Melleril or Melleryl or Mellaril or Melleretten or Melzine or Aldazine or Zuclopenthixol or alpha Clopenthixol or Cisordinol or Flufenazin* or Fluphenazine or Lyogen or Prolixin or ecopipam or Geodon or Seroquel or Haloperidol or quinolinone or Sch39166*).mp.

18. exp Anticonvulsants/

19. ((antiepileptic* or anti epileptic* or antiseizure* or anti seizure* or anticonvulsant* or anti convulsant* or anticonvulsive or anti convulsive*) and (agent* or drug* or intervention* or treatment* or pharmacotherap*)).ti,ab,kf.

20. (ACTH or (carbamazepine or Tegretol) or clorazepate or clobazam or clonazepam or chlordiazepoxide or divalproex or sodium divalproex or sodium valproate or (valproate or Depakote) or ethosuximide or ethosuccimide or ethotoin or felbamate or fosphenytoin or (gabapentin or Neurontin) or lignocaine or lamotrigine or Levetiracetam or lidocaine or hydantoins or levetiracetam or mephobarbital or methsuximide or oxcarbazepine or paraldehyde or phenacemide or phenytoin or pregabalin or primidone or succinimide or tiagabine or (topiramate or Topamax) or (valproate or Depacon) or vigabatrin or zonisamide).mp.

21. exp Valproic Acid/

22. exp "Serotonin and Noradrenaline Reuptake Inhibitors"/

23. exp Serotonin Uptake Inhibitors/

24. exp Antidepressive Agents/

25. exp Neurotransmitter Uptake Inhibitors/

26. (antidepress* or anti depress* or MAOI* or monoamine oxidase inhibit* or ((serotonin or serotonergic or norepinephrine or noradrenaline or nor epinephrine or nor adrenaline or neurotransmitt* or dopamine*) and (uptake or reuptake or reuptake)) or noradrenerg* or antiadrenergic or anti adrenergic or SSRI* or SNRI* or TCA* or tricyclic* or tetracyclic* or heterocyclic* or psychotropic*).mp.

27. (Agomelatine or Alaproclate or Amoxapine or Amineptine or Amitriptylin* or Amitriptylinoxide or Atomoxetine or Befloxatone or Benactyzine or Binospirone or Brofaromine or (Buproprion or Amfebutamone) or Butriptyline or Caroxazone or Cianopramine or Cilobamine or Cimoxatone or Citalopram or (Chlorimipramin* or Clomipramin* or Chlomipramin* or Clomipramine) or Clonidine or Clorgyline or Clovoxamine or (CX157 or Tyrima) or Demexiptiline or Deprenyl or (Desipramine* or Pertofrane) or Desvenlafaxine or Dibenzepin or Diclofensine or Dimetacrin* or Dosulepin or Dothiepin or Doxepin or Duloxetine or Desvenlafaxine or DVS233 or Escitalopram or Etoperidone or Femoxetine or Fluotracen or Fluoxetine or Fluvoxamine or (Hyperforin or Hypericum or St John*) or Imipramin* or Iprindole or Iproniazid* or Ipsapirone or Isocarboxazid* or Levomilnacipran or Lofepramine* or (Lu AA21004 or Vortioxetine) or Lu AA24530 or (LY2216684 or Edivoxetine) or Maprotiline or Melitracen or Metapramine or Mianserin or Milnacipran or Minaprine or Mirtazapine or Moclobemide or Nefazodone or Nialamide or Nitroxazepine or Nomifensine or Norfenfluramine or Nortriptylin* or Noxiptilin* or Opipramol or Oxaflozane or Paroxetine or Prazosin or Promazine or Phenelzine or Pheniprazine or Pipofezine or Pirlindole or Pivagabine or Pizotyline or Propizepine or Protriptylin* or Quinupramine or Reboxetine or Rolipram or Scopolamine or Selegiline or Sertraline or Setiptiline or Teciptiline or Thozalinone or Tianeptin* or Toloxatone or Tranylcypromin* or Trazodone or Trimipramine or Venlafaxine or Viloxazine or Vilazodone or Viqualine or Vortioxetine or Zalospirone).mp.

28. exp Alcohol Deterrents/

29. (Metadoxine or Tetrahydrocannabinol or Zofran or Pioglitazone or Aprepitant or Mecamylamine or Dutasteride or Ghrelin or Ivermectin or Isoflavone or Kudzu or Disulfiram or Metronidazole or Acamprosate or Propranolol or Doxazosin or Ketamine or Psilocybin or Agomelatine or Ondansetron or Varenicline or PUFAs or omega* or Oxytocin or Memantine or Citicoline or Diphenhydramine or Methylphenidate or Pexacerfont or Exenatide or Carisbamate or Perampanel or Flumazenil or Progesterone).mp.

30. exp Benzodiazepines/

31. exp benzodiazepinones/

32. (Benzodiazepin* or Adinazolam or Alprazolam or Bentazepam or Bretazenil or Bromazepam or Brotizolam or Camazepam or Chlordiazepoxide or Cinolazepam or Clobazam or Clonazepam or Clorazepate or Clotiazepam or Cloxazolam or Delorazepam or Devazepide or Diazepam or Estazolam or Etizolam or Fludiazepam or Flunitrazepam or Flumazenil or Flurazepam or Flutoprazepam or Halazepam or Haloxazolam or Ketazolam or Loflazepate or Loprazolam or Lorazepam or Lormetazepam or Medazepam or Metaclazepam or Mexazolam or Midazolam or Nimetazepam or Nitrazepam or Nordazepam or Oxazepam or Oxazolam or Phenazepam or Pinazepam or Prazepam or Premazepam or Propazepam or Quazepam or Ripazepam or Serazepine or Temazepam or Tetrazepam or Tofisopam or Triazolam or Zolazepam or Zaleplon or Zolpidem or Zopiclone).mp.

33. exp gamma-Aminobutyric Acid/

34. GABA agonist*.ti,ab,kf.

35. exp GABA agonists/

36. exp GABA Uptake Inhibitors/

37. (Baclofen or GHB or gamma Hydroxybutyric acid or gamma aminobutyric acid or sodium oxybate).mp.

38. exp Glutamatergic Agents/

39. (amantadin* or atomoxetin* or dcycloserin* or dextromethorphan or GLYX 13 or "MK 0657" or (ketamin* or Ketalar or Ketaject or Ketanest) or (lanicemin* or AZD6765) or memantin* or quinolin* or rellidep or riluzol* or (tramadol* or ETS6103 or viotra) or ampa or cerc 301 or d serin* or glun2b or glutamate or glutamin* or glutamatergic or glutathione* or glycin* or mglu* or N acetyl cysteine* or N methyl D aspartate or nmda or nrx 1074 or kainite or nr2b or sarcosin* or NAC).mp.

40. Calcium Channel Blockers/

41. (calcium adj3 (antagonist* or blocker* or inhibit*)).mp.

42. (amlodipine or amrinone or azelnidipine or bencyclan* or bepridil or AT877 or cilnidipine or cinnarizine or conotoxin* or daropidine or diltiazem or efonidipine or felodipine or fendiline or flunarizine or gallopamil or isradipine or lacidopine or lidoflazine or mibefradil or nicardipine or nifedipine or nimodipine or nisoldipine or nitrendipine or perhexiline or prenylamine or verapamil or magnesium sulph*).mp.

43. (therapy or drug therapy or rehabilitation).fs.

44. exp Drug Therapy/

45. or/9-44

46. randomized controlled trial.pt.

47. (randomi#ed or randomi#ation).ab,ti,kf.

48. RCT.ab.

49. (random* adj3 (administ* or allocat* or assign* or class* or control* or determine* or divide* or distribut* or expose* or fashion* or number* or place* or recruit* or subsitut* or treat*)).ab,kf.

50. placebo.ab,ti,kf.

51. trial.ab,ti,kf.

52. ((singl* or doubl* or tripl* or trebl*) adj3 (blind* or mask* or dumm*)).mp.

53. clinical trial, phase ii/ or clinical trial, phase iii/ or clinical trial, phase iv/ or randomized controlled trial/ or pragmatic clinical trial/

54. ((waitlist* or wait* list* or treatment as usual or TAU) adj3 (control or group*)).ab.

55. (((standard or routine or usual) adj2 (care or treatment or medication or therapy)) and (control* or group*)).ab.

56. or/46-55

57. psychotherap$.ti,ab,kf.

58. (psychotherap$ or psychoeducat* or psycho educat*).ti,ab,kf.

59. (behav* adj2 (activation or therap* or treat* or intervention or modification or train*)).ti,ab,kf.

60. (CBT or (cognitive adj2 (therap* or treat* or intervention or modification or train*))).ti,ab,kf.

61. (motivational adj2 (enhancement or interview or support or skills)).ti,ab,kf.

62. mindfulness.ti,ab,kf.

63. (famil* adj2 therap*).ti,ab,kf.

64. ((couple* or spouse* or partner* or marital or marriage or conjoint or interpersonal) adj2 (therap* or counsel* or treat* or intervention*)).ti,ab,kf.

65. (psycholog* adj2 (therap* or treat* or intervention or modification or train*)).ti,ab,kf.

66. exp Psychotherapy/

67. exp Self help Groups/

68. ((self adj2 help) and group*).ti,ab,kf.

69. (twelve adj2 step).ti,ab,kf.

70. exp Rehabilitation/

71. (group adj2 (activit* or discussion* or therap* or treat* or intervention* or support or train*)).ti,ab,kf.

72. problem solving.mp.

73. (psychosoci* or psycho soci* or social support).ti,ab,kf.

74. (volunteering or activity scheduling).ti,ab,kf.

75. (community adj2 (activit* or discussion* or therap* or treat* or intervention* or support or train*)).ti,ab,kf.

76. (contingency management or incentive* or reward or rewards or voucher* or money or monetary).ti,ab,kf.

77. ((alcohol* or addict*) adj2 (therap* or treat* or intervention or management or modification or support or train*)).ti,ab,kf.

78. or/57-77

79. 8 and (45 or 78) and 56

80. (random$ adj sampl$ adj7 ("cross section$" or questionnaire$1 or survey$ or database$1)).ti,ab. not (comparative study/ or controlled study/ or randomi?ed controlled.ti,ab. or randomly assigned.ti,ab.)

81. Cross-sectional study/ not (randomized controlled trial/ or controlled clinical study/ or controlled study/ or randomi?ed controlled.ti,ab. or control group$1.ti,ab.)

82. (((case adj control$) and random$) not randomi?ed controlled).ti,ab.

83. (Systematic review not (trial or study)).ti.

84. (nonrandom$ not random$).ti,ab.

85. "Random field$".ti,ab.

86. (random cluster adj3 sampl$).ti,ab.

87. (review.ab. and review.pt.) not trial.ti.

88. "we searched".ab. and (review.ti. or review.pt.)

89. (databases adj4 searched).ab.

90. (rat or rats or mouse or mice or swine or porcine or murine or sheep or lambs or pigs or piglets or rabbit or rabbits or cat or cats or dog or dogs or cattle or bovine or monkey or monkeys or trout or marmoset$1).ti. and animal experiment/

91. Animal experiment/ not (human experiment/ or human/)

92. exp animals/ not humans.sh.

93. or/80-92

94. 79 not 93
